# A missense mutation in *growth differentiation factor 9* (*GDF9*) is strongly associated with litter size in sheep

**DOI:** 10.1186/1471-2156-14-1

**Published:** 2013-01-02

**Authors:** Dag I Våge, Maren Husdal, Matthew P Kent, Gunnar Klemetsdal, Inger A Boman

**Affiliations:** 1 Centre for Integrative Genetics (CIGENE), Department of Animal and Aquacultural Sciences (IHA), Norwegian University of Life Sciences (UMB), PO Box 5003, N-1432, Ås, Norway; 2 The Norwegian Association of Sheep and Goat Breeders, PO Box 104, N-1431, Ås, Norway

## Abstract

**Background:**

A genome wide association study for litter size in Norwegian White Sheep (NWS) was conducted using the recently developed ovine 50K SNP chip from Illumina. After genotyping 378 progeny tested artificial insemination (AI) rams, a GWAS analysis was performed on estimated breeding values (EBVs) for litter size.

**Results:**

A QTL-region was identified on sheep chromosome 5, close to the *growth differentiation factor 9 (GDF9),* which is known to be a strong candidate gene for increased ovulation rate/litter size. Sequencing of the *GDF9* coding region in the most extreme sires (high and low BLUP values) revealed a single nucleotide polymorphism (c.1111G>A), responsible for a Val→Met substitution at position 371 (V371M). This polymorphism has previously been identified in Belclare and Cambridge sheep, but was not found to be associated with fertility. In our NWS-population the c.1111G>A SNP showed stronger association with litter size than any other single SNP on the Illumina 50K ovine SNP chip. Based on the estimated breeding values, daughters of AI rams homozygous for c.1111A will produce minimum 0.46 - 0.57 additional lambs compared to daughters of wild-type rams.

**Conclusion:**

We have identified a missense mutation in the bioactive part of the GDF9 protein that shows strong association with litter size in NWS. Based on the NWS breeding history and the marked increase in the c.1111A allele frequency in the AI ram population since 1983, we hypothesize that c.1111A allele originate from Finnish landrace imported to Norway around 1970. Because of the widespread use of Finnish landrace and the fact that the ewes homozygous for the c.1111A allele are reported to be fertile, we expect the commercial impact of this mutation to be high.

## Background

Litter size is an economically important trait in sheep breeding. To date, polymorphisms in three different genes have been associated with increased ovulation rate/litter size in sheep. These are the *growth differentiation factor 9 (GDF9)*, *bone morphogenetic protein 15 (BMP15)* and *bone morphogenetic protein receptor, type IB (BMPR1B)*.

The *BMP15* gene was first reported to be associated with increased ovulation rate in Hanna (FecX^H^) and Inverdale (FecX^I^) sheep [1]. In Hanna sheep a glutamine in position 291 was replaced by a premature stop codon (Q291X), while in the Inverdale sheep a valine residue in position 299 was replaced by aspartic acid (V299D). In the Merino Booroola sheep (FecB^B^), a glutamine to arginine mutation in position 249 (Q249R) of the *BMPR1B* gene was found to increase ovulation rate and litter size [2-4]. In Cambridge and Belclare sheep two additional mutations affecting ovulation rate were identified in *BMP15*, a substitution of glutamine with a premature stop codon in position 239 (Q239X) (FecX^G^) and a change of serine to isoleucine at position 367 (S367I) (FecX^B^) [5]. In the same study, a substitution of serine with phenylalanine in position 395 (S395F) (FecG^H^) of the *GDF9* gene was found to be associated with increased ovulation rate.

A common feature of the *GDF9* and *BMP15* mutations described above is that increased ovulation rate/litter size is observed for individuals being heterozygous for these mutations, while individuals being homozygous are sterile, indicating a dose dependent function of these proteins. Later, additional mutations with a similar effect have been identified in *BMP15*, a cysteine to tyrosine change in position 323 (C323Y) in Lacaune sheep (FecX^L^) [6] and a 17 basepair deletion in the Rasa Aragonesa sheep (FecX^R^) [7,8]. Also in *GDF9* an additional mutation was found, a substitution of serine with arginine in position 427 (S427R) (FecTT) [9].

In 2011 Silva et al., [10] reported a *GDF9* mutation, a substitution of phenylalanine with cysteine at position 345 (F345C) (FecG^E^). In contrast to previously reported mutations in both *GDF9* and *BMP15*, the F345C mutation in *GDF9* did not cause sterility in the homozygous individuals, but rather significantly higher prolificacy compared to heterozygous individuals. *GDF9* and *BMP15* are known to influence ovulation rate in a dose-responsive manner [11], indicating that the F345C variant has not completely lost its biological function.

In the present study we have used 378 rams selected for artificial insemination (AI rams) to search for polymorphisms affecting litter size in the Norwegian White Sheep (NWS) breed. All animals were included in a genome wide association study using the Illumina ovine 50K SNP chip. A QTL was detected on chromosome 5 which highlighted a previously detected candidate gene; *GDF9*. Sequencing of this gene in AI rams with extreme EBVs for litter size revealed a non-synonymous mutation in NWS.

## Results

378 AI rams were genotyped using Illumina's 50K ovine SNP array. Association testing was performed by a linear mixed model (GEMMA) [12], using estimated breeding values (EBVs) for daughter litter size as phenotypes. An initial test showed overlapping results when using EBV1, EBV2 and EBV3 as separate phenotypes (results not shown). We decided to use EBV1 phenotypes only for the association analysis, since this estimate is generated from the largest number of daughters compared to EBV2 and EBV3. Allowing for 5% missing data per SNP and 1% minor allele frequency, 47 986 SNPs were included in analysis.

**Figure 1:**
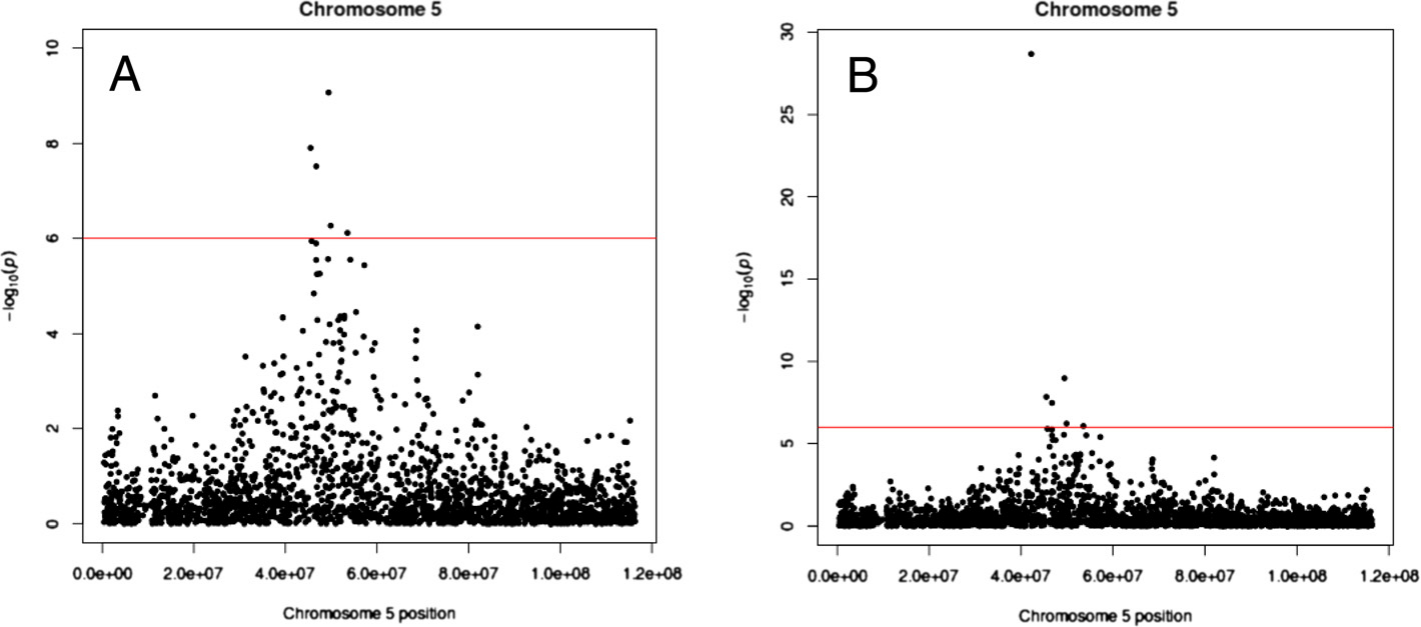
**Manhattan plot of ovine chromosome 5.** Mahattan plot showing SNPs associated with litter size on ovine chromosome 5. The chromosomal positions are shown in base pairs (bp) on the x-axis, while the –log_10_ of the likelihood ratio *p*-value is shown on the y-axis. The plot is shown without the *GDF9* c.1111G>A SNP included (**A**), and with *GDF9* c.1111G>A SNP included (**B**).

**Figure 2:**
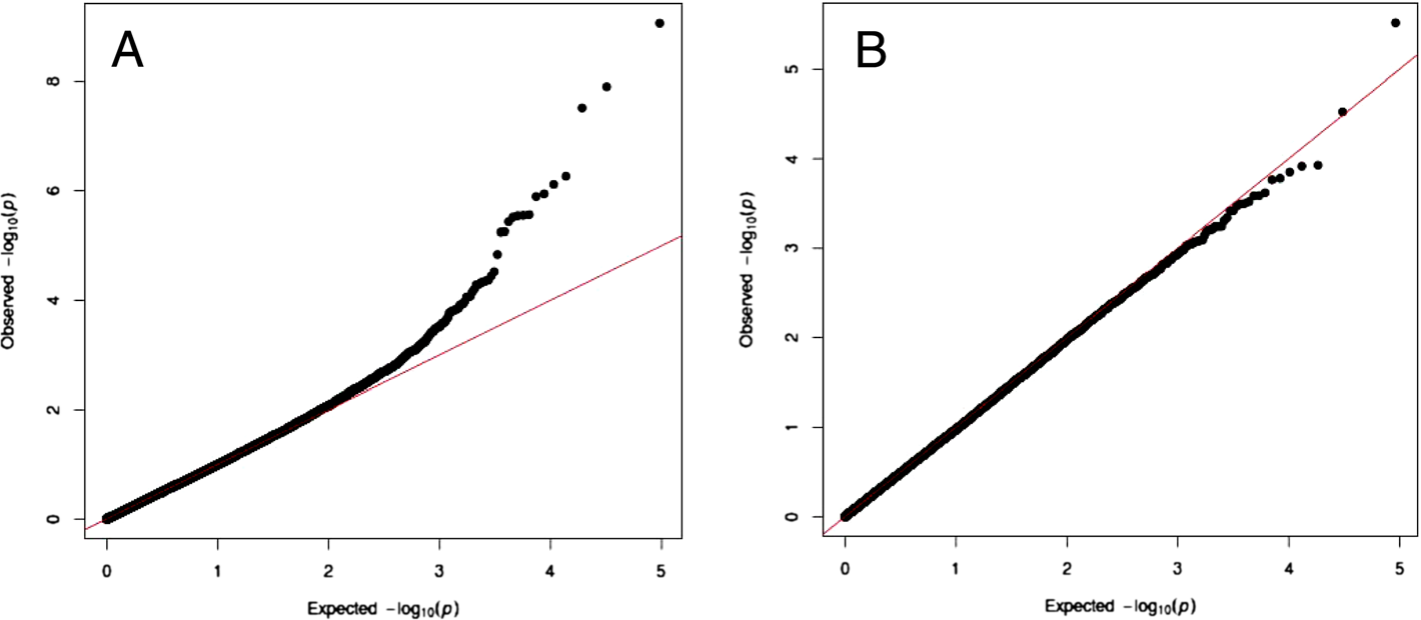
**QQ-plot of *p*-values without the *GDF9 *c.1111G>A SNP included.** Comparison of *p*-value distributions when all the 47 986 analyzed Illumina SNPs were included (**A**), and the corresponding plot when 2 112 SNPs on chromosome 5 were excluded (**B**). The expected –log_10_(*p*) is on the x-axis and the observed –log_10_(*p*) is on the y-axis.

Five SNPs on chromosome 5 showed significant association (*p* < 10^-6^), corresponding to a Boferroni corrected *p*-value < 0.05 (Table 1). A Manhattan plot of chromosome 5 is shown in Figure 1A, and the corresponding QQ-plot is shown in Figure 2A. To test for any remaining population stratification, an additional QQ-plot was made after removing chromosome 5 SNP *p*-values (Figure 2B), showing that the model is efficiently correcting for this.

**Table 1:** **Five Oar5 SNPs on the Illumina ovine 50K SNP array showing significant association with litter size, together with *GDF9 *c.1111G>A**

**SNP identity**	**Position at Oar v3.1**	**Unadjusted *p*-values**
s32463.1	45331371	8.607353e-10
OAR5_45481559.1	41841919	1.238006e-08
OAR5_46719933.1	42737138	3.030593e-08
OAR5_49873739.1	45779648	5.395519e-07
OAR5_53543443.1	49273490	7.651872e-07
*GDF9* c.1111G>A	41841345	2.122380e-29

Using the Bonferroni correction for multiple testing (*p*<0.05), five Oar5 SNPs on the Illumina ovine 50K SNP array showed significant association to litter size in Norwegian White Sheep. Chromosomal positions together with the unadjusted *p*-values are shown. The corresponding information for *GDF9* c.1111G>A is also shown.

**Figure 3:**
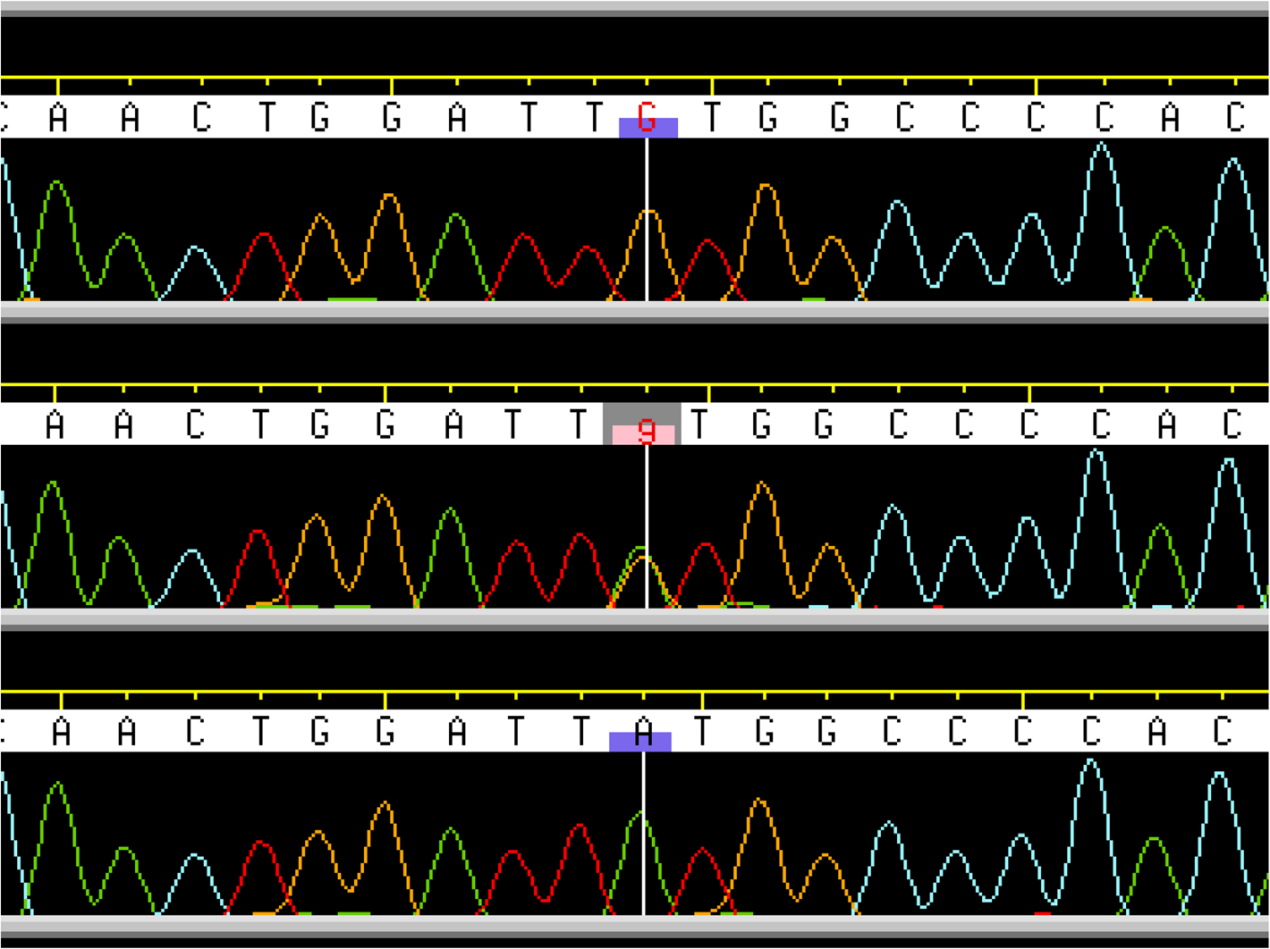
**A chromatographic representation of the c.1111G>A mutation.** The ovine *GDF9* c.1111G>A mutation. The figure show the sequence chromatograms from an individual homozygous for the G-allele (upper line), a heterozygous individual (GA) (middle line) and one individual homozygous for the A-allele (lower line). The sequences were assembled and viewed with Phred/Phrap/Consed software.

The location of these SNPs on chromosome 5, and proximity to a known candidate gene affecting litter size, *GDF9*, gave us reason to examine this sequence more closely. The two *GDF9* exons were sequenced (EMBL: HE866499) in 7 rams with high EBVs for litter size, and 6 rams with low EBVs, animals were chosen to be not closely related. This revealed one polymorphism where the alleles appeared to correlate with the litter size phenotype. This was a single nucleotide polymorphism (c.1111G>A) responsible for a Val→Met substitution at position 371 (V371M) (Figure 3). Except one heterozygous individual, 6 high fertility rams were homozygous for the A allele, while 6 low fertility rams were all homozygous for the G allele.

**Figure 4:**
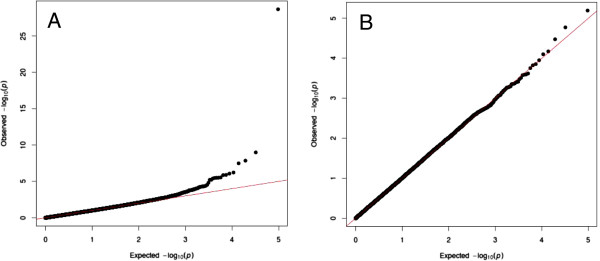
**QQ-plot of *p*-values with *GDF9 *c.1111G>A SNP included.** Comparison of *p*-value distributions when the *GDF9* c.1111G>A SNP is added to the dataset (**A**), and when the *GDF9* c.1111G>A SNP genotypes is included in the model as a covariate (**B**). The expected –log_10_(*p*) on the x-axis and the observed –log_10_(*p*) on the y-axis.

We subsequently repeated the GWAS analysis including the c.1111G>A SNP. The c.1111G>A SNP showed a -log_10_ value of 28.7 compared to 9.1 for the strongest associated SNP on the Illumina ovine 50K SNP array. A Manhattan plot including the c.1111G>A SNP is shown in Figure 1B, while the corresponding QQ-plot is shown in Figure 4A. A second QQ-plot was made including the c.1111G>A SNP genotypes as covariates (Figure 4B). These *p*-value distributions indicate that the deviation from the middle line is mainly explained by c.1111G>A SNP and its linked markers.

**Figure 5:**
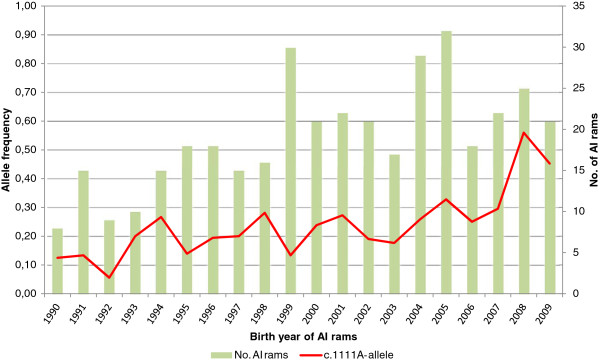
**Frequency changes of *GDF9*-alleles among the Norwegian White Sheep AI rams.** Frequency change in the *GDF9* allele c.1111A in NWS AI rams born in 1990-2009. The number of genotyped rams per birth year is presented by bars. The period 1983 – 1989 is omitted from the figure due to low numbers of rams per year (3 AI rams per year in average).

Genotyping the AI rams born from 1983 to 2009 for c.1111G>A gave an overall allelic distribution as follows: homozygous GG: 236 rams, heterozygous GA: 136 rams and homozygous AA: 31 rams. These results correspond to an overall allelic frequency of 0.25 and 0.75 of c.1111A and c.1111G, respectively. The c.1111A allele frequency has shown a tendency to increase from 1990 to 2009 as shown in Figure 5.

Genotype effects on litter size EBVs were highly significant, F-values were 102.84, 102.00 and 98.24 for EBV1, EBV2 and EBV3, respectively. Differences between least-squares means are given in Table 2. Sires homozygous for the mutation (AA) had an EBV that were 0.46 - 0.57 lambs higher than for the wild-type (GG), while the effect of the heterozygote (GA vs GG) was intermediate (0.20-0.25 lambs). These effects were consistently largest for the daughters at three years of age.

**Table 2:** **EBV contrasts between *GDF9 *c.1111 genotypes**

**Trait**	**GG vs GA**	**GA vs AA**	**GG vs AA**
EBV1	0.20	0.26	0.46
EBV2	0.22	0.29	0.51
EBV3	0.25	0.32	0.57

Effects of AI ram genotypes on daughters’ litter size given as least-squares means corrected for effect of birth-year. The effects are calculated from EBVs for litter size of daughters at one, two and three years of age (EBV1, EBV2 and EBV3, respectively). All differences shown are statistically significant (*p* < 0.0001).

## Discussion

By combining a genome wide association study and a candidate gene approach in AI rams of the Norwegian White Sheep breed, we successfully identified a SNP causing an amino acid change in the mature GDF9 protein. This SNP showed a stronger association to EBVs for litter size than any of the SNPs present at the Illumina 50K SNP array (Figure 1B). This sequence variant has previously been reported to exist in Belclare and Cambride sheep (the G7 polymorphism) [5], but these authors focused on polymorphisms that caused female sterility in the homozygous state. Animals homozygous for c.1111A were found to be fertile in that study, and no additional attempt to correlate this polymorphism with ovulation rate/litter size was performed. Also, the number of animals carrying this mutation was most likely too low to detect any genotype dependent variation in fertility.

**Figure 6:**
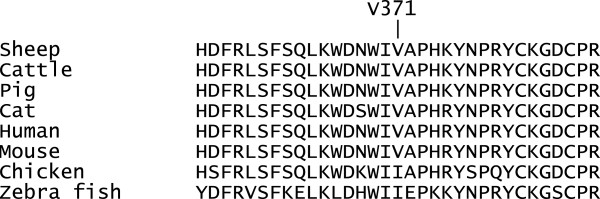
**Amino acid conservation at the 371 – position.** A sequence alignment comparing the protein sequence at ovine position 371 across Sheep (NP_001136360.1), Cattle (NP_777106.1), Pig (NP_001001909.1), Cat (NP_001159372.1), Human (NP_005251.1), Mouse (NP_032136.2), Chicken (NP_996871.2) and Zebra fish (NP_001012383.1).

Without any functional testing, we cannot conclude that the c.1111G>A is the causal mutation for the differences in EBVs for litter size observed in the NWS population, however our evidence is suggestive of a functional association for two reasons. Firstly, this is an amino acid change in the mature region (the bioactive part) of the GDF9 protein [13], and secondly, valine is found in this position across 6 highly different mammalian species (sheep, cattle, pig, cat, human and mouse), while in chicken and zebrafish valine is substituted by another aliphatic amino acid, isoleucine (Figure 6).

GDF9 is mediating its effect by binding to transforming growth factor, beta receptor 1 (TGFBR1) [14,15] and bone morphogenetic protein receptor, type II (BMPR2) [16]. As pointed out by Hanrahan et al. [5], the c.1111G>A polymorphism represents a relatively conservative change in the sense that one nonpolar amino acid (V) is substituted by another (M). However, the side chain of methionine is structurally different from that of valine, so depending on the location relative to the receptor binding region of GDF9, a reduced (but not lost) binding capacity can possibly be explained by this change.

Daughters of rams being homozygous for c.1111A gave birth to 0.46 - 0.57 additional lambs compared to daughters of c.1111G homozygous rams, while daughters of heterozygous rams gave 0.20 - 0.25 additional lambs. These figures can be considered as conservative, since they build on EBVs that are regressed towards the mean to compensate for a non-infinite number of progeny tested offspring. Based on the development of the c.1111A allele frequency in the AI rams over time (Figure 5), we can assume a similar but lagging frequency development in the ewe population, and thus low overall frequency. Basically, it will then be the heterozygous effect that is contained in the contrast of GG with AA rams, while the GA group with 50% heterozygote daughters will be logically intermediate. Also, when using the animal model for calculating EBVs without modeling the allele effect of c.1111A, the sire will mainly provide the allele, while the effect will be shared with the dam and therefore be underestimated when the frequency is low among the ewes.

The EBVs were estimated based on the daughters’ performance in terms of number of lambs born, but only ewes that gave births were included. Since GDF9 is known to influence oocyte maturation and ovulation rate, the ewe’s genotype will be determinative for her fertility. Therefore, this study should be followed up by genotyping a large number of ewes with known reproductive performance to obtain a better estimate of the separate genotypic effects, and to confirm that homozygous ewes are fertile as reported for Cambridge and Belclare sheep [5].

No single event in the breeding history of NWS has influenced litter size more than the introduction of Finnish landrace. Finnish landrace sheep are well known for their high fertility, and have been crossed to several breeds to investigate this trait [17,18]. Also in Norway, Finnish landrace was imported in late 1960s and early 1970s to improve fertility [19-21]. The significant phenotypic effect of the *GDF9* c.1111A - allele observed in this study could therefore indicate that this allele originate from Finnish landrace. The development in allele frequency in the AI rams population since 1990 also resemble the pattern observed for another imported allele, namely the Texel *MSTN* c.*1232A - allele [22]. In both cases a new breed has been imported to improve key traits, litter size and meatiness respectively, and after some latency the organized breeding system catches the causal allele and rapidly increase its frequency, first in the AI ram population and subsequently in the whole ewe population.

## Conclusions

We have identified a missense mutation in the bioactive part of the GDF9 - protein that is strongly associated with litter size in the NWS population. The observed amino acid change from valine to methionine might be the functional explanation of this trait, although the possibility of mutations outside the coding region in close linkage disequlibrium with c.1111G>A should not be excluded. According to the estimated breeding values, daughters of AI rams homozygous for c.1111A will produce minimum 0.46 - 0.57 additional lambs compared to daughters of rams not having this allele. Based on the breeding history of NWS and the recent increase in the allele frequency of c.1111A in this population, we hypothesize that this allele originates from imports of Finnish landrace. Because of the widespread use of Finnish landrace internationally, and the fact that this mutation is reported to also give fertile ewes in the homozygous state, we expect the commercial impact of this mutation to be high.

## Methods

### Animals

A total of 378 AI rams of the Norwegian White Sheep (NWS) breed, born between 1983 and 2008, were genotyped by the Illumina 50K SNP array and used for the GWAS - study. In addition to the 378 AI rams genotyped by the 50K SNP array, 21 AI rams born in 2009 and 4 rams born in 2007 and 2008 were genotyped for the *GDF9* c.1111G>A mutation and used for calculating allele frequencies. These additional 25 individuals were not included in the association analysis, nor in the analysis of genotype effects.

### Progeny testing and EBV calculations

All rams are progeny tested in so-called ram circles [19,23]. A ram circle consists of several farms that exchange rams during the breeding season to ensure that their offspring for progeny testing are born in different production environments (flocks). From 1991 the best linear unbiased prediction (BLUP) method and the animal model replaced the selection index approach for calculating breeding values. The phenotypic trait “number of lambs born” is referred to as litter size in this paper, and includes both live born and stillborn lambs. Separate BLUP EBVs for litter size are estimated, depending on whether daughters are one, two or three years of age. These data are referred to as EBV1, EBV2 and EBV3, respectively, and mean number of daughters per ram were 235, 212 and 148 respectively. Only ewes giving births are included in the EBV-calculation, leaving out sterile individuals. The phenotypic data used for prediction of breeding values was retrieved from the National Sheep Recording System (SRS), comprising all sheep born after 1987 in the ram circles.

### Genotyping by the ovineSNP50

DNA was isolated from semen using Qiagen MagAttract DNA extraction kit according to manufacturers protocols (Qiagen, Germany). DNA concentration was determined using PicoGreen reagent (Invitrogen, USA) and quality was assessed by gel electrophoresis using a 1% TAE agarose gel. Genotyping was performed using the OvineSNP50 array from Illumina (Illumina, USA) according to manufacturers recommendations. Raw data was converted to genotypes using the Illumina's Genotyping Module (version 1.9.4) within the Genome Studio Software (version 2011.1). Automatic clustering was performed with a call-threshold of 0.15, samples with call rates below 98% were excluded before performing manual re-clustering. SNP call frequency, minor allele frequency and pedigree error frequency were used as criteria to sort SNPs and facilitate manual adjustments. Of the 54 241 SNP assays present on the array, 79 were regarded as failing, with another 4 155 being monomorphic in our study.

### Association analysis

Estimated breeding values (EBVs) for litter size (measured as number-of-lambs-born when daughters are one year of age, i.e. EBV1) were used as phenotypes of the 378 genotyped AI rams included in the study. A linear mixed-model algorithm (GEMMA), that calculates exact values of standard test statistics, was used for the association analysis [12]. A standard relatedness matrix was estimated from SNP genotypes to account for population stratification and sample structure by this software. Only informative SNPs (> 95% data and > 1% MAF) were included in the study (n = 47 986). The significance of the associations was evaluated with likelihood-ratio test, and a conservative threshold for significance of *p *< 10^-6^ was applied using the Bonferroni correction for multiple testing, since (0.05/47986) = 1.04 × 10^-6^. The distribution of obtained versus expected genome-wide *p*-values were visualised by QQ-plots. The Manhattan plot and QQ-plots were made by a R-code provided at: http://gettinggeneticsdone.blogspot.no/2011/04/annotated-manhattan-plots-and-qq-plots.html.

### PCR amplification, cloning and sequencing of *GDF9*

Exon 1 of ovine *GDF9* was amplified by primer pairs 7916/7917, while exon 2 was amplified with primer pairs 7918/7919 and 7979/7980, respectively (Table 3). The primers were designed based on a genomic sequence that included both the *GDF9* exons (GenBank: AF078545.2 ). The exon 1 fragment was directly sequenced with primers 7916/7917, while the exon 2 fragment was directly sequenced with primers 7918/7919 and 7979/7980, using the BigDye® Terminator v3.1 kit (Applied Biosystems). Genomic DNA from 7 rams with high EBVs for litter size and correspondingly 6 rams with low EBVs were amplified and sequenced.

**Table 3:** **Primers used for amplification, sequencing and genotyping of the ovine *GDF9 *gene**

**Name**	**Direction**	**Position**	**Sequence5’-3’**
7916	Forward	1744 - 1763^a^	ATGGGGAAATGTGTTCCTTG
7917	Reverse	2187 - 2206^a^	CCACCCATTAACCAATCTGC
7918	Forward	3205 - 3224^a^	GGGGAGAAAAGGGACAGAAG
7919	Reverse	4283 - 4302^a^	GCCAGGACACTCATGGTTTT
7979	Forward	3863 - 3882^a^	AGGAGAGTGCCAGCTCTGAA
7980	Reverse	4443 - 4462^a^	CATGAGGAAGGCAGCTGTTA
*GDF9*F	Forward	3983 - 4002^a^	GCTTTAGTCAGCTGAAGTGG
*GDF9*R	Reverse	4039 - 4058^a^	CAGTCCCCTTTACAGTATCG
*GDF9*E	Extension	3996 - 4014^a^	GAAGTGGGACAACTGGATT

^a^Positions are numbered according to the genomic sheep *GDF9* sequence (Accession number: AF078545.2).

### Genotyping of the c.1111G>A polymorphism in ovine *GDF9*

The Sequenon massARRAY platform (SEQUENOM, San Diego, USA) was used for genotyping the c.1111G>A SNP according to manufacturers recommendations. Amplification primers (GDF9F and GDF9R) and the extension primer (GDF9E) are shown in Table 3. All animals included in the GWAS - study were genotyped by this assay, including those used in sequencing.

### Sequence alignment

To visualise the amino acid conservation across species at the sheep GDF9 371 position we used the COBALT program available at NCBI to align the following GDF9 protein sequences: Sheep (NP_001136360.1), Cattle (NP_777106.1), Pig (NP_001001909.1), Cat (NP_001159372.1), Human (NP_005251.1), Mouse (NP_032136.2), Chicken (NP_996871.2) and Zebra fish (NP_001012383.1).

### Genotype effects

All three genotypes (GG, GA and AA, respectively) were for the first time represented among the rams in 1994, and these and subsequently born rams up to 2008 (n = 319) were used to estimate the genotypic effect on EBVs. These 319 rams are a subset of the 378 rams genotyped by the 50K array. Rams born in 2009 have so far too few daughters that have lambed to be included. The following univariate linear model was applied:

(1) $${\mathrm{EBVi}}_{\mathrm{kl}}=\mu +{\mathrm{BY}}_{k}+G_{l}+e_{\mathrm{kl}}$$

where EBVi_*kl*_ = the estimated breeding value for number of lambs born of ewes at one, two and three years of age, for a ram born in year *k*, with genotype *l*; μ = overall mean of the trait; BY_*k*_ = the fixed effect of the *k*^th^ birth year of ram (1994-2008); G_*l*_ = the fixed effect of genotype *l* (GG, GA and AA, respectively) and e_*kl*_ is a random error term ~ *N*(0, *Iσ*_*e*_^2^), where I is the identity matrix and *σ*_*e*_^2^ is the residual variance. The birth year effect was included to account for significant (p < 0.001) genetic change over time, caused by other genes than the SNP in question.

## Competing interests

The authors declare that they have no competing interests.

## Authors’ contributions

DIV did the GWAS analysis, supervised the *GDF9* gene analyses and wrote up the first draft of the manuscript. MH sequenced *GDF9* and did the sequence analysis. MPK was responsible for the Illumina 50K genotyping and the subsequent quality analysis. GK contributed to the initiation of the study together with IAB and DIV and did the statistical analyses of the genotypic effects in collaboration with IAB. IAB provided the BLUP EBVs and also the phenotypic data for the GWAS analysis. All authors contributed to the writing of the paper, and have read and approved the final manuscript.
